# Investigation the Corrosion Inhibition Effect of Itraconazole on Copper in H_2_SO_4_ at Different Temperatures: Combining Experimental and Theoretical Studies

**DOI:** 10.3390/ma11112107

**Published:** 2018-10-26

**Authors:** Zhili Gong, Shini Peng, Xiaomei Huang, Lanzhou Gao

**Affiliations:** 1 School of Urban Construction and Environmental Engineering, Chongqing University, Chongqing 400044, China; snpeng_cqu@163.com (S.P.); xmhuang_cqu@163.com (X.H.); lzgao_cqu@163.com (L.G.); 2 School of Chemistry and Chemical Engineering, Yulin University, Yulin 719000, China

**Keywords:** Cu, itraconazole, acid corrosion inhibitor, morphology analysis, theoretical calculation, Langmuir adsorption model

## Abstract

The anti-corrosion inhibition effect of itraconazole on copper (Cu) in 0.5 M H_2_SO_4_ is observed with variety of experimental methods, including electrochemical measurement, surface morphology analysis, and theoretical calculations. These experimental results all confirm that itraconazole exhibits excellent anti-corrosion performance in the certain temperatures range (298 K–313 K) for copper in sulfuric acid solution. In addition, corresponding adsorption isothermal models were used to fit the adsorption behavior of itraconazole on the copper surface. The results show that the Langmuir adsorption model agrees best with the experimental results. The adsorption of itraconazole on the copper surface belongs to chemical and physical adsorption.

## 1. Introduction

The problems of metal materials corrosion and anti-corrosion are closely involve the development of modern science, technology and human life [1]. Metal materials are susceptible to corrosion by corrosive media during use, which affects their service life [2,3]. The phenomenon that metal properties generally degraded and destroyed over time called “corrosion” or “aging”. Metal corrosion not only causes certain economic losses, but also worsens the working environment. Therefore, the study of corrosion protection of metals has become a hot issue for anti-corrosion workers [4]. Among them, the addition of corrosion inhibitor is one of the most economical, convenient and practical means to inhibit metal corrosion [5,6,7].

Copper and its alloys are the earliest and most widely used non-ferrous metals in human applications, and the output ranks third in the world [8]. Due to its excellent thermal conductivity, mechanical properties and anti-fouling properties, it also has good strength, elasticity and wear resistance, so it plays an important role in various industrial fields [9,10]. In the power industry, the condensers of most power plants, the heat exchange devices of low-pressure heating and oil coolers, and the air-core conductors of water-cooled generators are all copper alloy materials. In the marine military industry, some copper alloy materials also used in the water pipeline systems of military warships such as aircraft carriers. However, due to the serious corrosion problem exposed during the use of copper materials, in the air, due to oxidation, the surface of copper and its alloys will form a relatively dense oxide film, the main components of which are CuO and Cu(OH)_2_. Therefore, the thermal conductivity and electrical conductivity of the copper alloy are seriously affected, which brings great harm to the product equipment, so the application of the copper product is greatly restricted. Fortunately, pickling can effectively remove the oxide film on the copper surface to obtain a bright copper surface. The commonly used pickling solution is sulfuric acid.

The addition of corrosion inhibitors to the acid wash solution not only availably inhibits the removal of the oxide film on the surface of copper, but also reduces the corrosion of the copper itself by the acid, which is of great significance [4]. Unfortunately, many corrosion inhibitor materials have excellent corrosion inhibition capabilities, but they are extremely limited in their application due to their serious environmental and human hazards [11]. Therefore, in recent years, many corrosion protection workers have begun to research and develop new corrosion inhibitors that are highly effective, low-toxic, and even non-toxic [12]. Tan et al. [13] and co-authors studied the inhibitory effect of the asthma drug montelukast sodium on copper in H_2_SO_4_. Itraconazole can be orally and used as the corrosion inhibitor due to low concentrations. Therefore, the harm to the human body and the environment is very small. We drew on Tan’s work and studied the corrosion inhibition performance of the antifungal drug itraconazole on pure copper in sulfuric acid. As is well known, corrosion inhibitor molecules usually contain heteroatoms such as nitrogen, oxygen, sulfur, etc., and there are solitary electron pairs in these heteroatoms [14,15,16,17,18], which can form coordination bonds with the empty orbitals of the metals of transition to form a dense protective film, thereby the corrosive medium is isolated effectively from the metal surface for protection. Itraconazole contains a large number of heteroatoms and conjugated rings, so it can be used as a potential inhibitor.

## 2. Experimental Section

### 2.1. Materials Preparation

**Figure 1:**
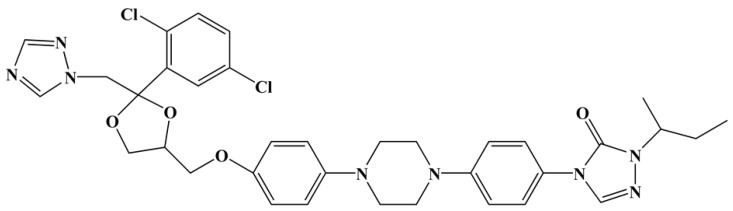
Chemical structure of the itraconazole.

Itraconazole was purchased from Hasen Pharmaceutical Co., Ltd., Shanghai, China. Its molecular formula is presented in Figure 1.

The corrosion solution of 0.5 M sulfuric acid was prepared using high purity concentrated sulfuric acid and ultrapure water. Then, itraconazole were dissolved in the 0.5 M H_2_SO_4_ to prepare the test solution of 0.04, 0.2, 1, and 5 mM, respectively.

The pure copper electrode is fabricated using the following process. First, the copper block was cut into a square with a side length of 1 cm using a linear cutter. Next, rough grinding on 180-mesh metallographic sandpaper. Finally, the other five sides of the copper electrode were sealed in a PVC (Polyvinylchlorid) tube with epoxy, leaving only one side exposed to the corrosive medium.

### 2.2. Surface Investigation by Scanning Electron Microscope, Atomic Force Microscope

Prior to testing the SEM, the researched pure copper block was cut into three cubes with a side length of 0.3 cm. Sanding on 400–7000 mesh sandpaper in turn until the testing surface is flat. Two copper cubes were separately immersed in 0.5 M H_2_SO_4_ in the absence and presence of 5 mM itraconazole for 24 h. The scanning electron microscope made in Japan (JEOL Ltd., Tokyo, Japan) and its model number is JEOL-JSM-7800F.

Prior to testing the AFM, the pure copper block was cut into three cubes with a side length of 0.2 cm. The copper samples were treated in the same manner as the SEM described above. This atomic force microscope made in the USA (Asylum Research, Santa Barbara, CA, USA) and its model number is MFP-3D-BIO.

### 2.3. Electrochemical Experiments

All electrochemical experiments were carried out in the three-electrode system. The entire three-electrode system was placed in a 150 mL beaker. The testing solution guaranteed to be 100 mL at each experiment, ensuring complete immersion of the three-electrode system. In this three-electrode system, the working electrode is the pure copper electrode. Prior to each test, the copper electrode was sanded on 180–2000 mesh sandpaper until the surface to be tested was flat and bright. The counter electrode was a square platinum plate electrode (1 cm^2^). The reference electrode is a saturated calomel electrode connected using a glass of Luggin capillary.

All electrochemical tests were performed on the chi760E electrochemical workstation (Shanghai Chenhua Co., Ltd., Shanghai, China). The electrochemical experiments consist of three parts. In the first step, the open circuit potential of this system was tested and the test time was 3000 s to ensure that the potential fluctuation range was within 3 millivolts during the last 300 s. The stable value of the open circuit potential (*E_OCP_*) was recorded. The second step was to conduct electrochemical impedance spectroscopy. The corresponding parameter settings are as follows: Init E is the *E_OCP_*, high frequency is 100,000 Hz, and low frequency is 0.01Hz, the value of the amplitude is 0.005 V and the quiet time is 2 s. Finally, the potentiodynamic polarization curve test was performed. The polarization range was *E_OCP_* − 250 mV to *E_OCP_* + 250 mV with a scanning rate of 1 mV/s. All experiments of the same conditions were performed three times to obtain good reproducible results.

### 2.4. Theoretical Calculation

The molecular properties of itraconazole were calculated using Material Studio software 8.0 with the DMol3 module. The relevant calculation parameters were set as follows: the calculation task is geometry optimization, quality was fine, functional was B3LYP, and Spin unrestricted and use formal spin as the initial parameters were set. The molecular structure of itraconazole was optimized, and its Highest Occupied Molecular (HOMO) and Lowest Unoccupied Molecular (LUMO) molecular orbitals and dipole moment were calculated.

The adsorption of the itraconazole molecule on the Cu (111) side was simulated using the Forcite module in the Material Studio software. The entire calculation process was as follows: First, cut the Cu (111) face, then set a 5 × 5 × 5 super cell and a vacuum layer. After fixing the positions of all the copper atoms, the vacuum layer was filled with an itraconazole molecule and 500 water molecules. After the filling was completed, the structure of the entire system was optimized, and then the entire system as subjected to molecular dynamics simulation. The parameters are set as follows: COMPASS (Condensed-phase Optimized Molecular Potentials for Atomistic Simulation Studies) is used as the force field, the time step is 1 fs, and the total simulation time is 500 s.

## 3. Results and Discussion

### 3.1. Blank Solution Electrochemical Analysis at Different Temperatures

**Figure 2:**
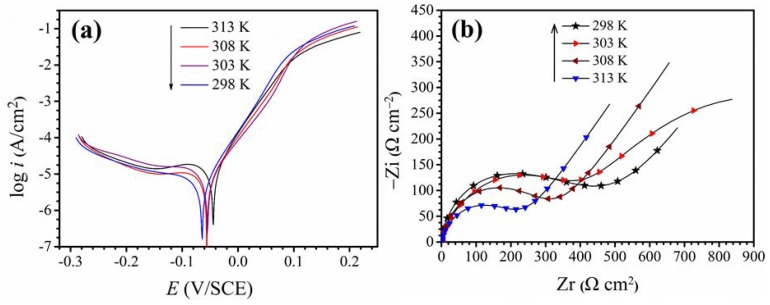
(**a**) The plot of potentiodynamic polarization of copper in 0.5 mol/L H_2_SO_4_ without itraconazole and (**b**) the impedance spectra at disparate temperatures.

The potentiodynamic polarization curves of copper in 0.5 M sulfuric acid show in Figure 2a at different temperatures. It can be clearly seen from the Figure 2a that the corrosion current density increases with the temperature increases, and the corrosion voltage moves toward the anode. Figure 2b shows the impedance spectrum of the copper in 0.5 M H_2_SO_4_. From Figure 2b, it can be seen that the radius of the capacitive arc decreases with increasing temperature. This indicates that the charge transfer resistance (*R_ct_*) decreases as the temperature increases, so it can be proved that the increase in temperature exacerbates the corrosion of copper. In addition, it can be found that the Warburg impedance appearing at low frequency increases significantly with increasing temperature. Typically, the Warburg impedance is due to the diffusion of corrosion products from the electrode surface into the bulk solution or the dissolved oxygen in the solution diffuses to the surface of the research electrode [4,13,19]. Obviously, when the temperature of the corrosion solution rises, the movement speed of the dissolved oxygen in the solution is increased, and it is easy to get rid of the attraction of the solvent molecules. Therefore, dissolved oxygen is not the factor that affects the Warburg impedance values to increase. However, the kinetic energy of the corrosive medium in the solution increases significantly after the temperature rises, which accelerates the corrosion of the copper electrode. Leading more copper ions diffuse into the bulk solution. Therefore, the Warburg impedance values increase with increasing temperature [20].

### 3.2. Polarization Curves

**Figure 3:**
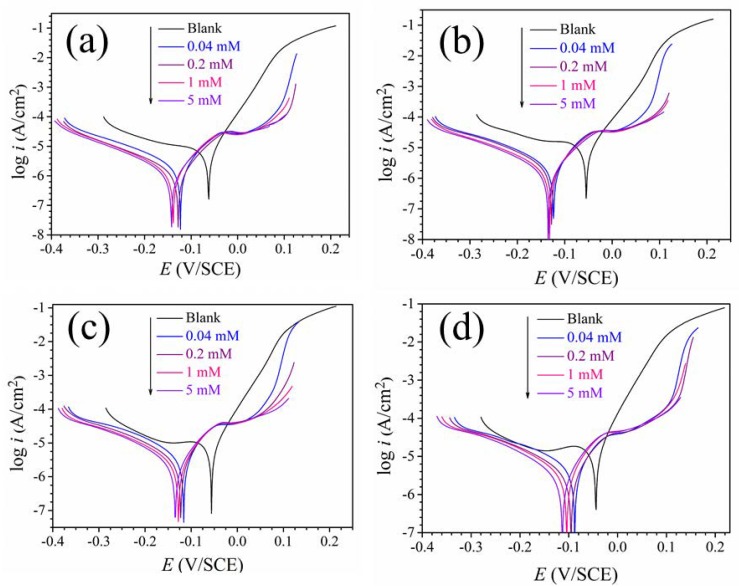
Polarization plots of Cu at different temperatures in 0.5 mol/L H_2_SO_4_ with and without itraconazole: (**a**) 298 K, (**b**) 303 K, (**c**) 308 K, and (**d**) 313 K.

In order to study the corrosion inhibition process, the plots of potentiodynamic polarization curves to copper in the 0.5 mol/L H_2_SO_4_ without itraconazole at different temperatures are shown in Figure 3. Some important parameters, including corrosion current density (*I_corr_*), corrosion potential (*E_corr_*), etc., can be obtained by Tafel extrapolation. These polarization parameters are presented in Table 1, where the corrosion inhibition efficiency (*η*) is obtained by the corresponding corrosion current density, as shown in Equation (1) [21,22,23,24]:

(1) $$\eta (\%)=\frac{I_{corr,0}-I_{corr}}{I_{corr,0}}\times 100\;$$

where *I_corr_*_,0_ and *I_corr_* correspond to the corrosion current density in the blank solution and the presence of itraconazole, respectively.

It can be seen from Figure 3a that as the concentrations of itraconazole increases, the corrosion current density moves to the lower direction. Corrosion current density is 18.2 μA·cm^–2^ in the absence of itraconazole. The corrosion current density drops sharply to 0.78 μA·cm^–2^ when the concentration of itraconazole reaches 5 mM. Furthermore, it can be clearly seen from Figure 3 that as the polarization potential increases, the slope of the polarization curves in the anode region increase significantly at the high concentration of itraconazole. This phenomenon indicates that the corrosion inhibitor molecules have desorbed on the surface of the copper electrode [4]. In addition, it can be clearly found that, in Figure 3a, the magnitude of the decrease in the cathodic polarization curve is significantly larger than that of the anode after the addition of the itraconazole molecules. It is indicated that the addition of itraconazole molecules effectively inhibits the reduction of dissolved oxygen near the surface of the electrode. Observing the trend of the change in corrosion potentials, it can be found that as the concentration of itraconazole increases, the corrosion potentials move in the negative direction. By observing Table 1, the value of moving to the negative electrode at different temperatures is slightly less than 85 mV, In addition, it can be seen from Figure 3 that the tendency of the cathode branch corrosion current density to decrease is significantly faster than that of the anode branch. Which indicates that the inhibition of the cathode is significantly larger than that of the anode. Therefore, it can be judged that itraconazole is a mixed-type corrosion inhibitor which mainly inhibit the cathode reaction [25].

The mechanism of copper corrosion in acid solution is as follows. The reaction mechanism of the anode and cathode a as follows [13,26,27,28]:

Anodic reaction:

$$Cu_{s}\to \;{\mathrm{Cu}}_{\left(ads\right)}^{+}+1e^{-}\;\mathrm{fast}\;$$

$$Cu_{\left(ads\right)}^{+}\to Cu_{\left(sol\right)}^{2+}+1e^{-}\;\mathrm{slow}\;$$

Cathodic reaction:

$$\frac{1}{2}O_{2}+2H^{+}+2e^{-}\to H_{2}O\;$$

From the corrosion mechanism of the anode, it can be found that the process of oxidizing the copper substrate to cuprous ion is very fast. Therefore, many corrosion workers believe that the inhibition mechanism of the corrosion inhibitor on copper is as follows [29,30]:

$$Cu^{+}+inhibitors\to Cu^{+}:inhibitors\;$$

Therefore, the corrosion inhibitor molecule is more easily combined with cuprous ions to form a coordination bond, which effectively isolates the corrosive medium from the copper substrate, thereby achieving a good corrosion inhibition effect.

By observing Figure 3 and Table 1, it can be found that with the increase of temperature, the inhibition effect of itraconazole on Cu in H_2_SO_4_ is slightly reduced. Still, at 313 K, the inhibition efficiency of itraconazole on Cu in 0.5 mol/L H_2_SO_4_ is still as high as about 90%. Therefore, it can be concluded that itraconazole exhibits excellent corrosion inhibition performance in a certain temperature range for Cu in H_2_SO_4_.

### 3.3. Electrochemical Impedance Spectroscopy Test

**Figure 4:**
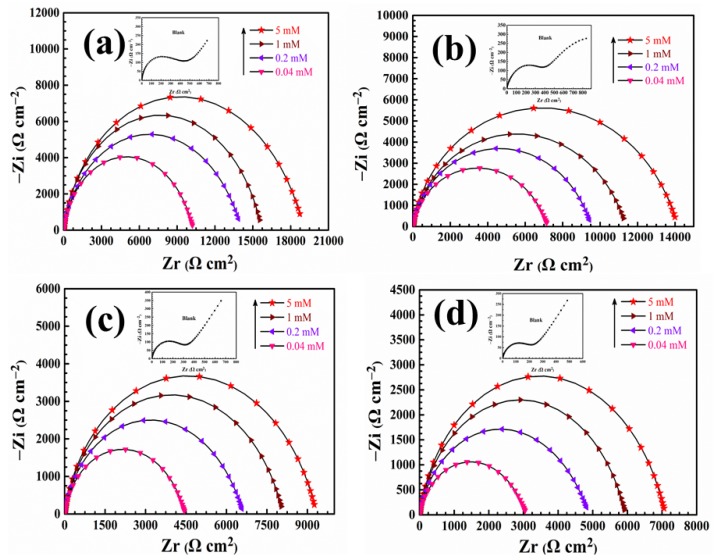
Nyquist plots of copper present and lacking itraconazole in 0.5 mol/L H_2_SO_4_ at various temperatures: (**a**) 298 K, (**b**) 303 K, (**c**) 308 K, and (**d**) 313 K.

Electrochemical impedance spectroscopy (EIS) experiment was executed study the inhibitory effect of itraconazole on copper at different temperatures and the kinetics of the corrosion reaction process. The Nyquist plots of the copper in 0.5 mol/L H_2_SO_4_ without and with different concentrations of itraconazole are shown in Figure 4.

As shown in Figure 4, the plots of Nyquist in the blank solution at different temperatures consists of a straight line at the low frequency area (Warburg impedance) and a capacitive reactance arc at the high frequency area (typically related to the electric double layer capacitance and charge transfer resistance). The low frequency line means that the copper is corroded in 0.5 M H_2_SO_4_. That is, the corrosion products of the Cu surface diffuse into the bulk solution. Therefore, the slope of the line in the low frequency area increases with the temperature increases, which means that the copper corrosion is more serious.

As the concentration of itraconazole increases, the straight line at the low frequency area disappears, which proves that the corrosion product of the copper surface diffuses into the bulk solution effectively suppressed. In addition, the apparent increase in the radius of the capacitive reactance is attributed to an increase in the charge transfer resistance of the surface of the copper electrode. These indicate that itraconazole forms a dense resistant film on the surface of Cu to effectively inhibit copper corrosion. Besides, the capacitive reactance arc emerges imperfect semicircles, which are related to the heterogeneity of the copper surface [31]. When the temperature of the corrosion system increases, we can find that the corrosion inhibition performance of itraconazole is reduced, which is consistent with the experimental results of potentiodynamic polarization.

**Figure 5:**
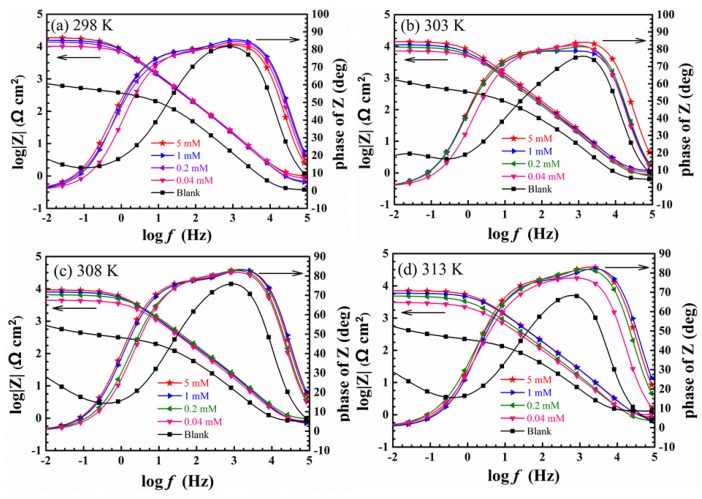
Bode plots of copper present and lacking itraconazole at various temperatures in 0.5 M H_2_SO_4_: (**a**) 298 K, (**b**) 303 K, (**c**) 308 K, and (**d**) 313 K.

Figure 5 presents Bode plots of copper present and lacking itraconazole molecules at various temperatures in 0.5 mol/L H_2_SO_4_. As the concentration of itraconazole increases, the impedance modulus increases almost two orders of magnitude in the low frequency region at different temperatures comparison to the blank solution. Moreover, the curves of the phase angle value become significantly larger and wider. In Figure 5, it can be clearly seen that two distinct peaks appear in the phase angle, whose represent the presence of two time constants in the presence of itraconazole. This indicates that the itraconazole molecules have two relaxation processes in the adsorption process on the copper surface. One relaxation process is the surface electric double layer capacitance of the copper electrode, and the other relaxation process is the itraconazole molecule adsorption on the copper surface [32]. This similar Bode plots change trend is observed at diverse temperatures in Figure 5.

**Figure 6:**
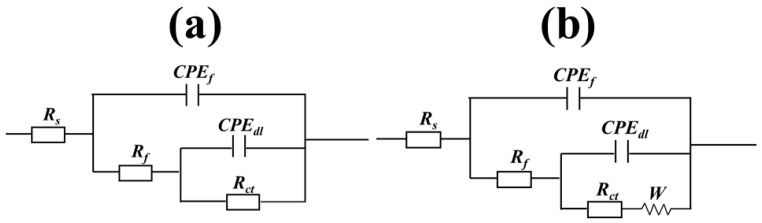
The equivalent circuit diagrams required to fit the impedance spectrum data: (**a**) fit the equivalent circuit diagram containing the itraconazole molecule, and (**b**) fit the equivalent circuit diagram in the blank solution.

The two equivalent circuits were used to fit the equivalent electrochemical impedance data, as shown in Figure 6. The errors between in experimental data and fitting results are measured by chi-square values (χ^2^). The chi-square values after fitting in this study are all equal to or less than 10^−3^. The fitted electrochemical parameter values are listed in Table 2. *R_s_* represents the solution resistance and *R_f_* is the film resistance. *R_ct_* indicates that the charge transfer resistance is attributed to the corrosion reaction at the copper-solution interface. *W* is the Warburg impedance, *CPE_f_* is the constant phase angle element which consists of adsorbed inhibitor film capacitance *C_f_* and the deviation parameter *n*_1_. *CPE_dl_* which consists of an electric double layer capacitor *C_dl_* and the deviation parameter *n*_2_. The Equation (2) of the CPE impedance is as follows [33,34,35]:

(2) $$Z_{CPE}=\frac{1}{Y_{0}{\left(j\omega \right)}^{n}}\;$$

where *Y*_0_ represents the admittance electrochemical system, *j* represents the imaginary root, *ω* stands for the angular frequency, *n* is the deviation parameter. The values of *C_f_* and *C_dl_* are calculated with Equation (3) by *ω*, *n* and *Y*_0_ as following [4]:

(3) $$C=Y_{0}{\left(\omega \right)}^{n-1}=Y_{0}{\left(2\pi f_{Z_{im\text{-}Max}}\right)}^{n-1}\;$$

where $f_{Z_{im\text{-}Max}}$ is the frequency corresponding to the maximum imaginary impedance.

In addition, according to the Helmholtz model, According to the Helmholtz model, the Equations of *C_dl_* and *C_f_* are as follows [36,37]:

(4) $$C_{dl}=\frac{\varepsilon^{0}\varepsilon }{d}S\;$$

(5) $$C_{f}=\frac{F^{2}S}{4RT}\;$$

where *d* represents the distance between itraconazole and metal surface. *S* is the copper area exposed in the H_2_SO_4_ solution, *ε*^0^ is the permittivity of vacuum, *ε* is the local dielectric constant. *F* stands for the Faraday’s constant. According to Equations (4) and (5), it can be found that the decrease in the *C_dl_* and *C_f_* values are ascribed the itraconazole molecules replace the H_2_O molecules on the copper surface.

The most important indicator the inhibition efficiency is calculated according to Equation (6) as follows:

(6) $$\eta (\%)=\frac{R_{ct}-R_{ct,0}}{R_{ct}}\times 100\;$$

wherein *R_ct_* and *R_ct_*_,0_ are the charge transfer resistance in the presence and absence of itraconazole in H_2_SO_4_. Obviously, as the temperature and the concentration of itraconazole increase, the corrosion inhibition efficiency increases. This conforms highly to the potentiodynamic polarization test results.

### 3.4. SEM Surface Investigate

**Figure 7:**
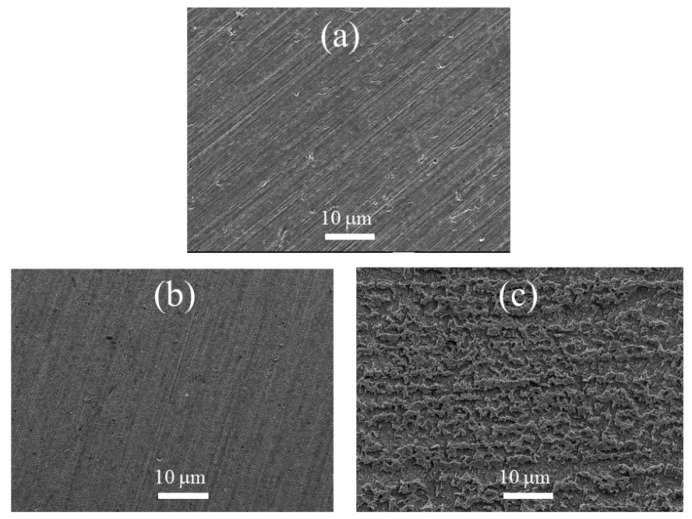
Surface feature of copper in the different condition: (**a**) the surface of the copper after the sanding, (**b**) the copper after the addition of the inhibitor molecule is immersed in 298 K at 0.5 mol/L H_2_SO_4_ for 24 h, and (**c**) copper surface after soaking for 24 h in 0.5 mol/L H_2_SO_4_ in 298 K.

Figure 7 shows the feature of the copper surfaces at different conditions. Figure 7a is the feature of the copper surface after grinding. Figure 7b shows the surface feature at 298 K after soaking for 24 h in 0.5 M H_2_SO_4_ with 5 mM itraconazole. Figure 7c shows the feature of copper surface immersed in 0.5 M H_2_SO_4_ at the temperature of 298 K for 24 h without any corrosion inhibitor. It can be found that the bare copper had obvious nicks and lines after polishing as shown in Figure 7a, due to the copper left behind after grinding the SiC sandpaper. Figure 7c clearly shows the pores left by the corrosion of copper in 0.5 M H_2_SO_4_. These corrosion holes are very uniform, but the copper surface is severely corroded. Figure 7b shows the tight protective film barrier formed by the studied itraconazole on the copper surface. In addition, after immersing in sulfuric acid for 24 h on the copper surface, it is still flat and uniform. No obvious corrosion holes are found, and some nicks left after polishing can still be seen. Therefore, by comparing the SEM maps of Figure 7b,c, it indicates that the addition of 5 mM itraconazole in the 0.5 M sulfuric acid solution can effectively inhibit copper corrosion.

### 3.5. AFM Surface Analysis

**Figure 8:**
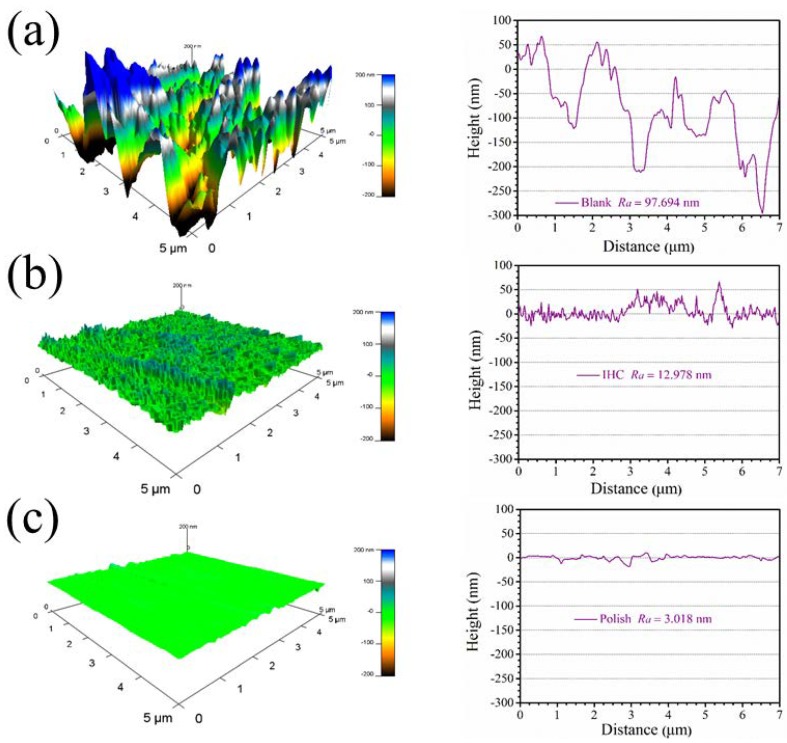
3D topography of copper under different conditions; (**a**) copper sample soaked in 0.5 mol/L H_2_SO_4_ for 24 h without itraconazole and the 2D topography image on the diagonal. (**b**) The copper sample was soaked in 0.5 M sulfuric acid for 24 h containing 5 mM itraconazole and the 2D topography image on the diagonal, and (**c**) the freshly polished copper surface and the 2D topography image on the diagonal.

Atomic force microscopy has been widely applied in the research of corrosion inhibitors, and the surface topography structure information and surface roughness information are obtained due to nanometer resolution. In this study, in order to more deeply observe the 3D topography information of the copper surface, the topography of the copper surface under different conditions was tested. Figure 8a shows, from left to right, the 3D topography of copper immersed in 0.5 mol/L H_2_SO_4_ for 24 h and the 2D topography image on the diagonal. The average roughness of the entire surface is as high as 97.694 nm. Figure 8b shows, from left to right, the 3D topography of copper after soaking in 0.5 M sulfuric acid for 24 h in 5 mM itraconazole and the 2D topography image on the diagonal. Obviously, the average roughness of the entire surface after the addition of itraconazole acutely dropped to 12.978 nm, and the protective film of itraconazole on the copper surface was also observed on the entire surface. Figure 8c shows the 2D topography image of the polished copper surface and the diagonal line from left to right. After sanding, the scratches of the sandpaper leave a certain scratch. The average roughness is 3.018 nm. By comparing the surface topography image of copper under different conditions, it can visually demonstrate the excellent corrosion inhibition performance of itraconazole.

### 3.6. Adsorption Isotherm Research

In order to gain a deeper understanding of the adsorption mechanism of the itraconazole molecules studied on the copper surface, the polarization curve data and various adsorption isotherms were used to fit, respectively, Langmuir (7) [38,39,40,41], Temkin (8) [42], El-Awady (9) [42], Flory-Huggins (10) [43,44], and Frumkin (11) isotherm equations. Their corresponding formula expressions are as follows:

Langmuir isotherm equation:

(7) $$\frac{C}{\theta }=\frac{1}{K_{ads}}+C$$

Temkin adsorption isotherm:

(8) $$\exp^{\left(-2\alpha \theta \right)}=KC$$

Flory-Huggins adsorption isotherm:

(9) $$\ln\frac{\theta }{C}=x\ln(1-\theta )+\ln(xK_{ads})$$

El-Awady adsorption isotherm:

(10) $$\ln\frac{\theta }{1-\theta }=y\ln C+\ln K^{\prime }$$

Frumkin adsorption isotherm:

(11) $$\ln[\frac{\theta }{(1-\theta )C}]=\ln K+2\alpha \theta$$

**Figure 9:**
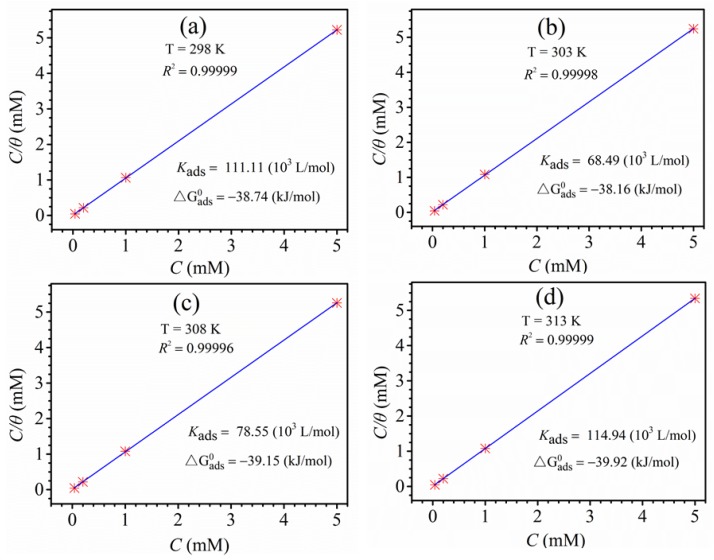
The Langmuir isotherm equation plots of copper with different concentrations of itraconazole in 0.5 mol/L H_2_SO_4_ at diverse temperatures.

The all the corresponding fitted results are shown in Figures S1–S4, and Figure 9, and the fitted results show that the regression coefficients (*R*^2^) of the other four isothermal equations are smaller than the Langmuir isotherm equation. Therefore, only the Langmuir isotherm equation has the best relationship with the experimental data. The regression coefficient (*R*^2^) of the Langmuir isotherm equation exceeds 0.9999 at different temperatures. In the Langmuir isotherm equation, *K_ads_* is the equilibrium adsorption constant, and the standard $\Delta G_{ads}^{0}$ free energy can be calculated with *K_ads_*. The expression is as follow [45,46]:

(12) $$K_{ads}=\frac{1}{55.5}\exp(-\frac{\Delta G_{ads}^{0}}{RT})\;$$

where *R* stands for the molar gas constant and *T* stands for the thermodynamic temperature.

The thermodynamic parameters of the itraconazole molecule adsorbed on the copper surface obtained from the Langmuir adsorption isotherm at different temperatures are also shown in Figure 9. Corrosion workers generally believe that smaller values of $\Delta G_{ads}^{0}$ and larger *K_ads_* indicate that the inhibitors studied can be densely and stably adsorbed on metal surfaces [47]. It can be seen from Figure 9 that the value of *K_ads_* is higher at different temperatures and the value of $\Delta G_{ads}^{0}$ is relatively small. Therefore, itraconazole can have a perfect corrosion inhibition ability for copper. In addition, the values of $\Delta G_{ads}^{0}$ are −38.74 kJ/mol, −38.16 kJ/mol, −39.15 kJ/mol, and −39.92 kJ/mol at 298, 303, 308, and 313 K, respectively. It can be clearly found that is very close to −40 kJ/mol at different temperatures. Therefore, it can be considered that the itraconazole molecule is mainly chemically adsorbed on the copper surface with a small amount of physical adsorption [19].

### 3.7. Quantum Chemical Calculation Analysis

**Figure 10:**
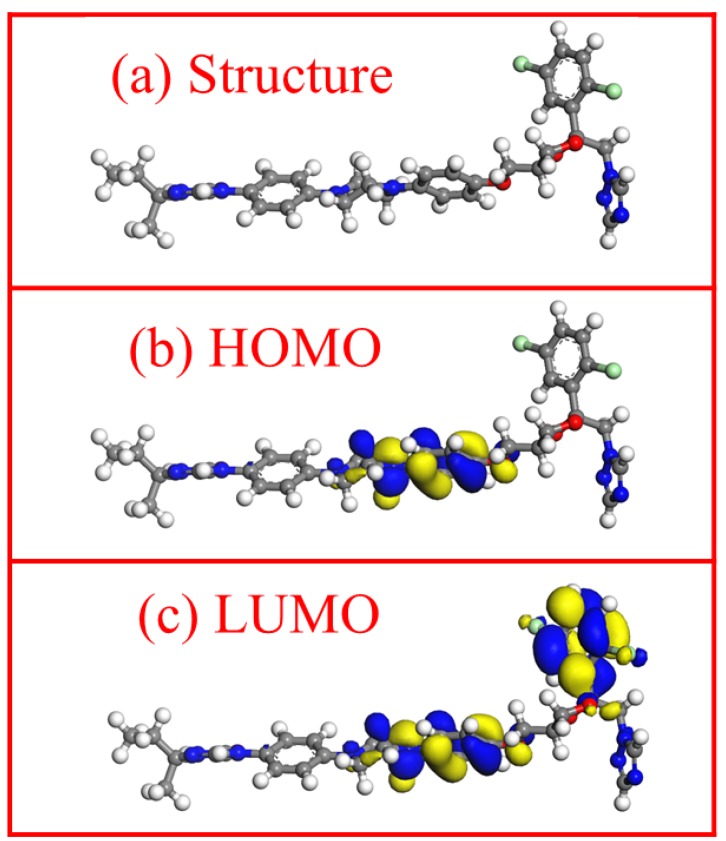
Itraconazole molecule after optimization (**a**), the HOMO orbital (**b**), and the electron cloud distribution of the LUMO orbital (**c**).

Quantum chemical calculations are widely applied study the properties of corrosion inhibitor molecules. An understanding of corrosion inhibitors can be further enhanced by calculating some important quantitative parameters of the corrosion inhibitor molecules. In this paper, we separately optimized the molecular structure of itraconazole, and calculated the frontier molecular orbital and dipole moment by MS software 8.0. Figure 10 shows the optimized structural formula of itraconazole, the electron cloud distribution of HOMO and LUMO, and calculates the energy of the HOMO and LUMO molecular orbitals of the itraconazole molecule.

According to the frontline orbital theory of Japanese chemist Fukui Kenichi: the highest energy molecular orbital HOMO is related to the electron donating capacity of the molecule [48]. The *E_HOMO_* value of itraconazole is 4.11 eV. This higher value means that the itraconazole molecule has a strong electron donating nature, and the electrons given are easily combined with cuprous ions to develop a stable adsorption film on the copper surface, thereby effectively inhibiting copper corrosion. Conversely, the lowest energy molecular orbital LUMO is related to the electron accepting nature of the molecule. The energy gap (Δ*E* = *E_LUMO_* − *E_HOMO_*) is usually discussed as a key parameter when discussing the molecular properties of corrosion inhibitors. A low Δ*E* value corresponds to a higher corrosion inhibition efficiency [19]. Itraconazole has a Δ*E* value of 2.41 eV, which means that itraconazole exhibits excellent corrosion inhibition.

### 3.8. Molecular Dynamics Simulation

**Figure 11:**
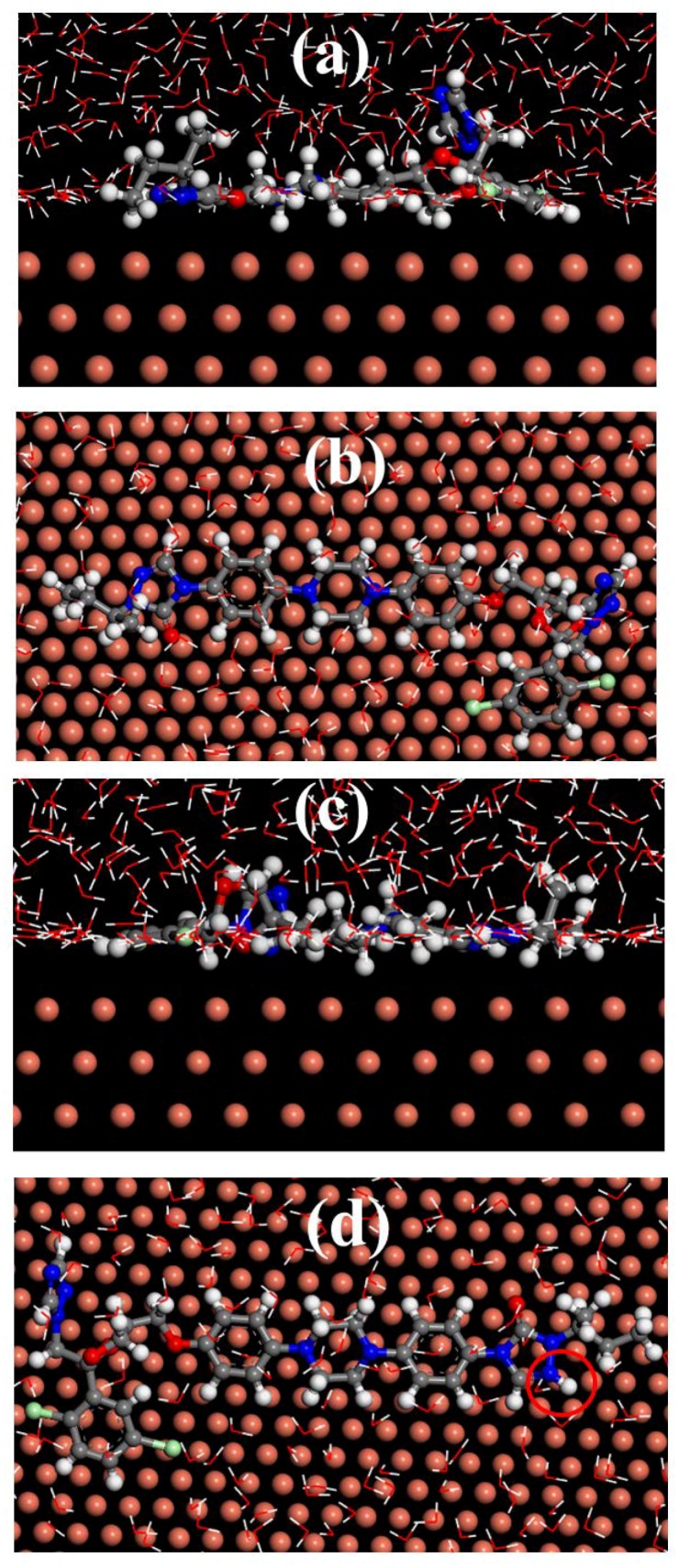
The stable configuration of itraconazole and itraconazole after protonation adsorbed on Cu (111) surface: (**a**) side view plot of itraconazole, (**b**) top view plot of itraconazole, (**c**) side view plot of itraconazole itraconazole after protonation, and (**d**) top view plot of itraconazole after protonation.

Molecular dynamics simulation (MDS) can calculate the adsorption configuration of inhibitor molecules on the metal surface, and can determine the adsorption strength of corrosion inhibitor molecules on the metal surface by the binding energy of adsorption. Figure 11 shows the stable adsorption configurations of the itraconazole and itraconazole after protonation on the Cu surface. It can clearly find that itraconazole and itraconazole after protonation adsorbed on Cu surface via a nearly parallel manner, thus effectively inhibiting the corrosion of the copper substrate by the corrosive medium. However, by comparing Figure 11a–c, it can be seen that itraconazole is more parallel adsorbed on the copper surface after protonation in the acid solution. The adsorption binding energy of itraconazole on the copper surface can be obtained by the following Equations [4,49,50,51]:

(13) $$E_{interact}=E_{tot}-(E_{subs}+E_{inh})\;$$

(14) $$E_{binding}=-E_{interact}\;$$

Among them, *E_tot_* is the energy of the entire simulation system including an itraconazole molecule, 500 water molecules and a copper substrate for research. *E_subs_* is the energy of the copper base and *E_inh_* is the energy of the research molecule. The binding energy values of itraconazole and itraconazole after protonation are calculated to as high as 1017 kJ/mol and 1067 kJ/mol via Equations (11) and (12), respectively. High binding energy value indicates that corrosion inhibitor corresponds to good corrosion inhibition properties. The binding energy value indicates that the protonation of itraconazole is beneficial to the adsorption of copper surface in the acid solution. Furthermore, it can be clearly proved that itraconazole has excellent retardation on the copper surface, and the experimental results are highly consistent.

## 4. Conclusions

(1) Electrochemical experiments show that itraconazole is a mixed corrosion inhibitor for Cu in H_2_SO_4_. The corrosion inhibition efficiency can still be maintained at about 90% within a certain temperature range.
(2) The SEM and AFM images demonstrate that the itraconazole molecules form a compact film on the copper surface to effectively inhibit copper corrosion.
(3) Itraconazole molecules adsorb on the Cu surface conforms to the Langmuir adsorption isotherm model and is dominated by chemisorption.
(4) Theoretical calculations show that itraconazole adsorbs on the surface of Cu via a parallel manner.

## Figures and Tables

**Table 1:** Polarization parameters of itraconazole molecule at different temperatures in copper in 0.5 M H_2_SO_4_.

*T* (K)	*C* (mM)	*E_corr_* (mV/SCE)	*I_corr_* (μA·cm^−2^)	*β_c_* (mV·dec^−1^)	*β_a_* (mV·dec^−1^)	*η* (%)
	Blank	62	18.20	121.4	41.4	-
	0.04	123	1.91	136.8	61.4	89.5
298	0.2	128	1.36	159.1	62.6	92.5
	1	138	1.09	127.6	50.2	94.0
	5	142	0.78	112.5	62.4	95.7
	Blank	55	21.71	209.5	49.1	-
	0.04	124	2.15	154.4	57.8	90.1
303	0.2	128	1.76	156.2	55.7	91.9
	1	132	1.26	135.3	66.4	92.2
	5	135	1.01	106.8	63.7	95.3
	Blank	56	25.31	109.6	47.0	-
	0.04	116	2.68	163.3	51.9	89.4
308	0.2	123	2.12	157.2	56.9	91.6
	1	128	1.86	157.5	63.6	92.6
	5	135	1.23	150.8	66.2	95.1
	Blank	44	28.64	113.8	46.9	-
	0.04	88	3.65	200.8	70.9	87.3
313	0.2	95	2.98	164.1	72.9	89.6
	1	104	2.15	156.9	61.9	92.5
	5	113	1.82	151.1	54.1	93.6

**Table 2:** Fitting EIS parameters of the Cu in 0.5 mol/L H_2_SO_4_ at different temperatures with and without different concentrations of itraconazole.

*T* (K)	*C* (mM)	*R_s_* (Ω·cm^2^)	*R_f_* (Ω·cm^2^)	*R_ct_* (kΩ·cm^2^)	*C_f_* (μF·cm^−^^2^)	*n*_1_	*C_dl_* (μF·cm^−2^)	*n*_2_	*W* (×10^−2^ Ω·cm^2^·s^1/2^)	*η* (%)
	Blank	1.31	0.9	0.55	60.4	0.49	68.9	1	1.19	-
	0.04	0.89	150.1	10.14	49.8	0.97	32.7	0.73	-	94.6
298	0.2	0.76	170.6	13.90	40.2	1	31.5	0.72	-	96.0
	1	1.21	232.3	15.40	35.7	0.96	29.7	0.76	-	96.4
	5	1.35	329.4	18.94	28.9	0.98	27.8	0.74	-	97.1
	Blank	1.24	8.3	0.37	65.2	0.41	71.5	0.59	4.10	-
	0.04	1.76	71.94	7.01	51.3	0.95	34.5	0.59	-	94.7
303	0.2	2.12	112.5	9.42	46.5	1	31.9	0.74	-	96.1
	1	3.15	140.2	11.13	38.9	0.97	28.8	0.75	-	96.7
	5	2.49	231.2	13.62	27.8	0.99	26.5	0.76	-	97.3
	Blank	1.39	13.2	0.31	68.9	0.54	74.2	0.65	8.02	-
	0.04	1.61	96.13	4.37	59.7	0.97	36.5	0.71	-	92.9
308	0.2	1.87	153.4	6.47	51.2	0.94	33.1	0.70	-	95.2
	1	1.65	161.1	7.95	43.7	1	27.9	0.75	-	96.1
	5	1.76	180.8	9.16	31.3	0.98	24.3	0.76	-	96.6
	Blank	1.27	25.2	0.23	72.2	0.49	77.6	0.55	10.37	-
	0.04	2.07	31.72	3.05	60.3	1	34.5	0.68	-	92.4
313	0.2	1.89	80.28	4.82	56.8	0.98	29.6	0.67	-	95.2
	1	2.45	120.1	5.83	48.7	0.96	26.5	0.75	-	95.7
	5	1.88	174.6	6.92	34.2	1	22.8	0.76	-	96.7

## Supplementary Materials

The following are available online at http://www.mdpi.com/1996-1944/11/11/2107/s1, Figure S1: The Flory-Huggins adsorption isotherm plots of copper with different concentrations of itraconazole in 0.5 mol/L H_2_SO_4_ at diverse temperatures, Figure S2: The Temkin adsorption isotherm plots of copper with different concentrations of itraconazole in 0.5 mol/L H_2_SO_4_ at diverse temperatures, Figure S3: The El-Awady adsorption isotherm plots of copper with different concentrations of itraconazole in 0.5 mol/L H_2_SO_4_ at diverse temperatures, Figure S4: The Frumkin adsorption isotherm plots of copper with different concentrations of itraconazole in 0.5 mol/L H_2_SO_4_ at diverse temperatures.
