# Supplementary material for: Investigation the Corrosion Inhibition Effect of Itraconazole on Copper in H2SO4 at Different Temperatures: Combining Experimental and Theoretical Studies
Source: Materials (Basel). 2018 Oct 26;11(11):2107. doi: 10.3390/ma11112107 (PMC6265726; doi:10.3390/ma11112107)
Supplement: Supplementary file 1 [file materials-11-02107-s001.pdf]

materials

MDPI

Article

Investigation the Corrosion Inhibition Effect of Itraconazole on Copper in H_2SO_4 at Different Temperatures: Combining Experimental and Theoretical Studies

Zhili Gong [1,2,*], Shini Peng [1], Xiaomei Huang [1] and Lanzhou Gao [1]

[1] School of Urban Construction and Environmental Engineering, Chongqing University, Chongqing 400044, China; snpeng_cqu@163.com (S.P.); xmhuang_cqu@163.com (X.H.); lzgao_cqu@163.com (L.G.)
[2] School of Chemistry and Chemical Engineering, Yulin University, Yulin 719000, China
* Correspondence: zlgong_cqu@163.com

Received: 12 September 2018; Accepted: 25 October 2018; Published: date

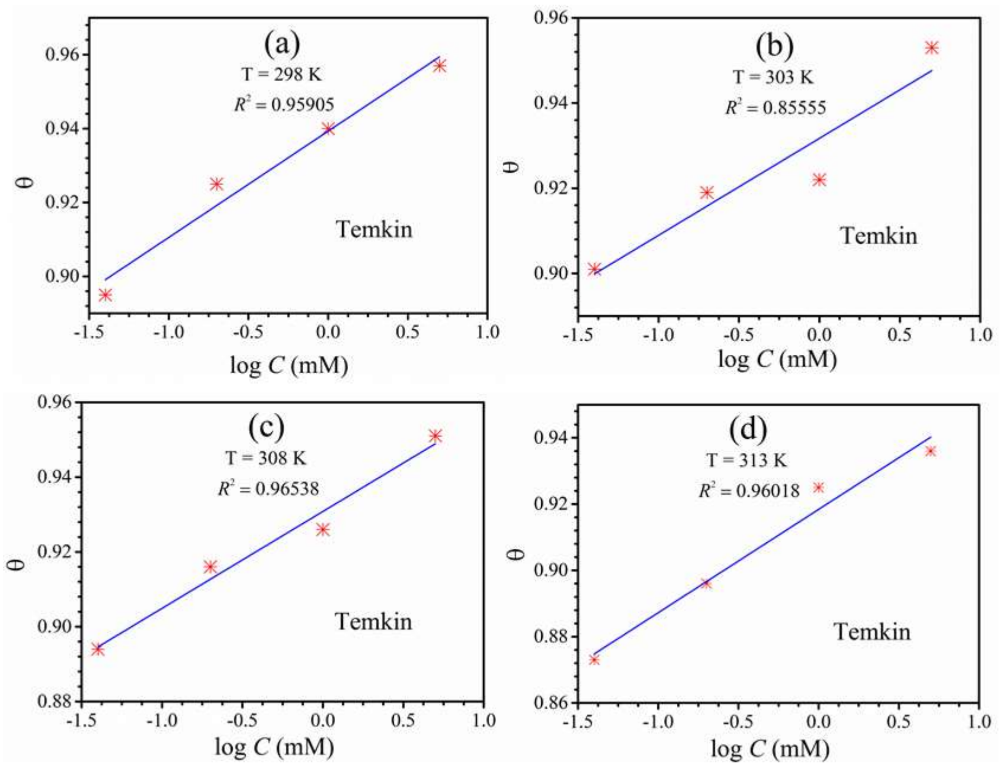

Figure S1. The Flory-Huggins adsorption isotherm plots of copper with different concentrations of itraconazole in 0.5 mol/L H_2SO_4 at diverse temperatures.

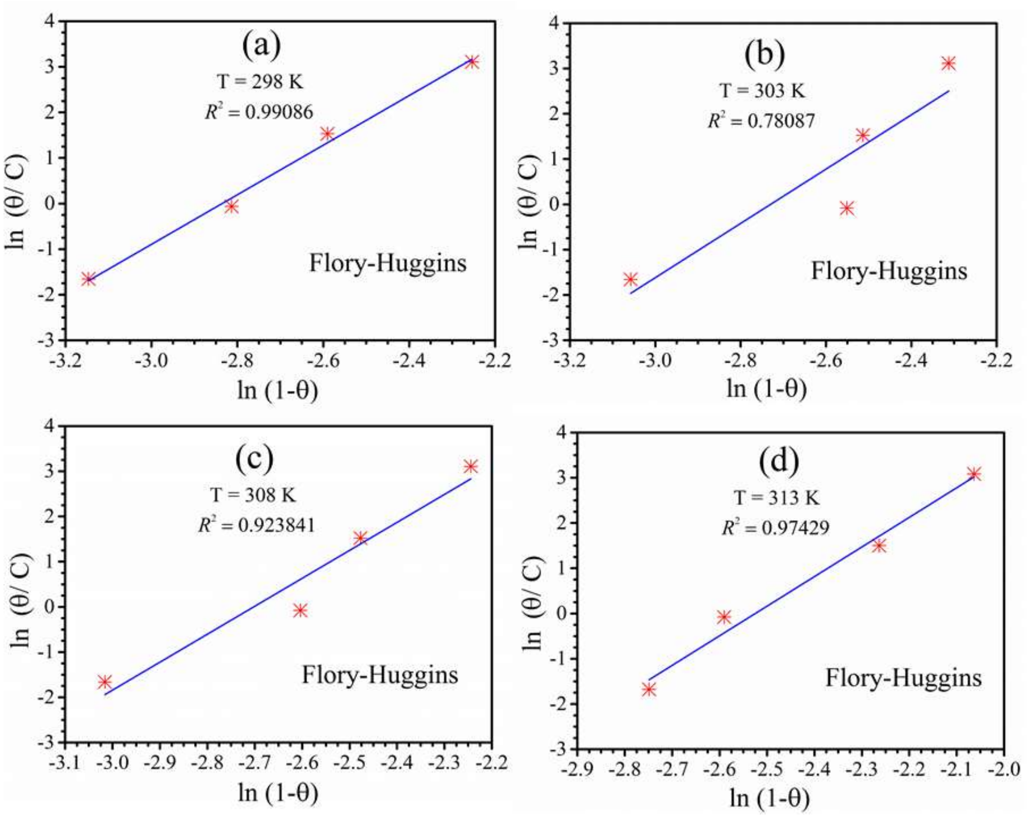

Figure S2. The Temkin adsorption isotherm plots of copper with different concentrations of itraconazole in 0.5 mol/L H_2SO_4 at diverse temperatures.

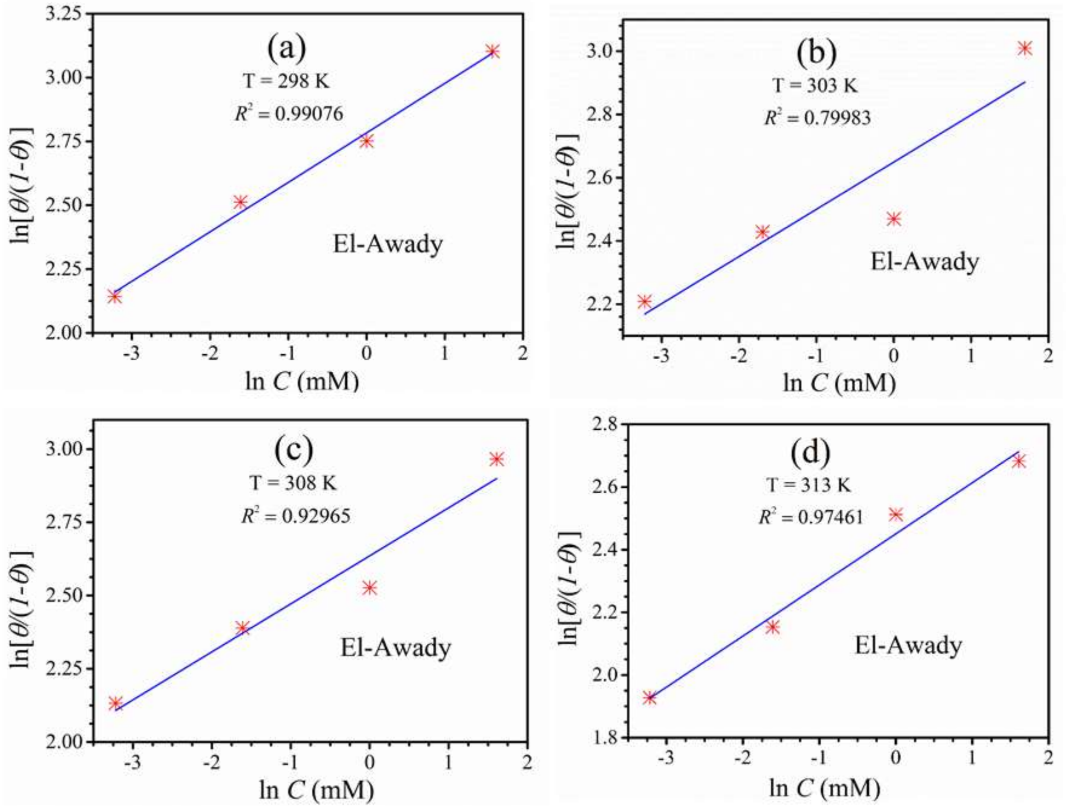

Figure S3. The El-Awady adsorption isotherm plots of copper with different concentrations of itraconazole in 0.5 mol/L H_2SO_4 at diverse temperatures.

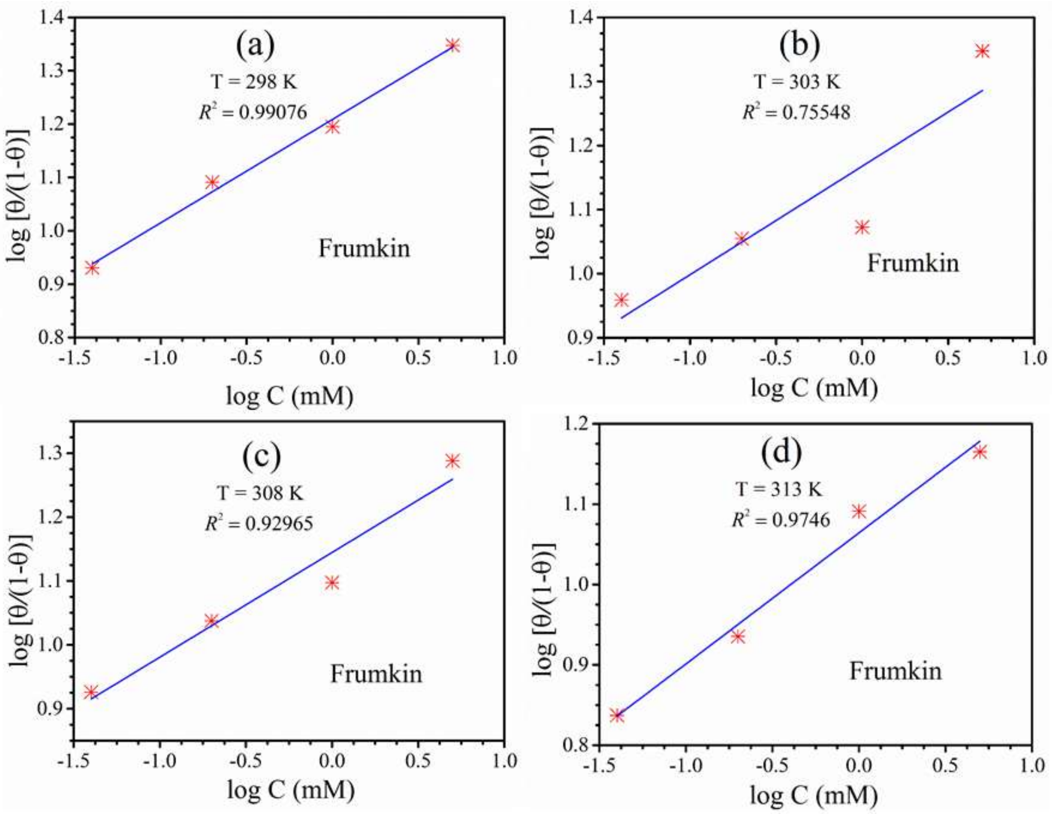

Figure S4. The Frumkin adsorption isotherm plots of copper with different concentrations of itraconazole in 0.5 mol/L H_2SO_4 at diverse temperatures.

© 2018 by the authors. Submitted for possible open access publication under the terms and conditions of the Creative Commons Attribution (CC BY) license (http://creativecommons.org/licenses/by/4.0/).
